# Dental Status of New Caledonian Children: Is There a Need for a New Oral Health Promotion Programme?

**DOI:** 10.1371/journal.pone.0112452

**Published:** 2014-11-07

**Authors:** Hélène Pichot, Martine Hennequin, Bernard Rouchon, Bruno Pereira, Stéphanie Tubert-Jeannin

**Affiliations:** 1 Clermont University, University of Auvergne, EA 4847, Centre de Recherche en Odontologie Clinique, BP 10448, Clermont-Ferrand, France; 2 Sanitary and Social Agency of New Caledonia, Nouméa, New Caledonia; 3 CHU Clermont-Ferrand, Clermont-Ferrand, France; University of Washington, United States of America

## Abstract

**Background:**

Before implementing a new oral health promotion program in the French overseas territory of Nouvelle Calédonie, the health authorities needed recent data about dental status of the New Caledonian child population.

**Objectives:**

This study aimed to describe the dental status of 6, 9 and 12-yr-old New Caledonian children and to investigate the environmental and behavioural risk factors related to oral health.

**Methods:**

A randomly selected sample of 2734 children (744 6-yr-olds, 789 9-yr-olds, and 1201 12-yr-olds) was examined clinically by seven calibrated investigators and participants responded to a questionnaire. The main variables were objective criteria about dental status and subjective criteria about experience of dental care, dental fear, self-perception of oral health, cultural or ethnic identity and environmental and behavioural risk factors.

**Results:**

Overall, most of the children had infectious oral diseases: more than 50% had gingivitis, and 60% of 6- and 9 yr-olds had at least one deciduous or permanent tooth with untreated caries. The mean 12-yr-old number of decayed missing and filled teeth (DMFT) was 2.09±2.82. The number of carious lesions was related to the unfavourable lifestyle, deprived social status and no preventive dental care. Kanak, Polynesians and Caledonians (respectively 27%, 18% and 45% of the study sample) were more affected by caries than metropolitan French and Asian children. Children with many untreated carious lesions had negative perceptions of their oral health; they complained of chewing difficulty and had higher scores for dental anxiety.

**Conclusion:**

This study highlights the need for new strategies aimed at improving oral health and at reducing inequalities in New Caledonia. An oral health promotion program would need to be developed in connection with other health programmes using the common risk factor approach within the context of the local environment.

## Introduction

Although oral health has improved in many industrialised countries, the World Health Organization (WHO) recently emphasised that dental caries remains a major health problem in disadvantaged and poor population groups in both developing and developed countries [1]. New Caledonia (pop. 245 580 persons) is a French overseas territory with extensive administrative autonomy. The population is a mix of 40% Kanak people (the original inhabitants of New Caledonia), 30% White European people (Caledonians and Metropolitan Frenchmen), 10% Polynesian people (Wallisians essentially), South-East Asian people and people from Vanuatu. There is little in the literature about the oral health of the New Caledonian population. The last national study was conducted in 1996 and concerned the dental status of 12-yr-old children only [2]. Furthermore, this national study only reported DMF scores and no other aspects of children's dental status. Since then, some local studies have been conducted but they had methodological flaws and did not include adults or other child age groups.

Considering the lack of information about children's dental status in 2011, the New Caledonian health authorities required more complete and recent oral health data. Several aspects of New Caledonia had to be considered during the conception of this study. In New Caledonia, health and educational services are administrated separately in three regions: the North, the Islands and the South. The population varies according to the region, with more Kanak people in the North and the Islands and more Polynesians, white Europeans and Asians in the South. Three quarters of the population live in the South, where economic activity is concentrated, and 39% of the total population lives in Nouméa, the administrative capital. The mean income per inhabitant places New Caledonia within the richest countries of the South Pacific area but there are strong social and economic disparities within the population. The aim of this study was to provide epidemiological data on the dental status of a representative sample of 6-, 9- and 12-yr-old children in New Caledonia. To provide a more comprehensive view of oral health, we also explored the relationships existing between dental status, health behaviours and socioeconomic status (SES) and the children's perceptions of their oral health and dental anxiety.

## Population and Methods

### Study population

The study population included children aged 6, 9 and 12 years (born in 2005, 2002 and 2000), who were pupils in New Caledonian schools between July 2011 and September 2012.

### Sample size

The desired sample size was calculated using the formula N = 1.96×P(1-P)/i2 with dental caries prevalence (P) as the main indicator, a precision (i) of 5% according to a previous study from New Caledonia [4] and the first species risk (α) was fixed at 0.05. Caries prevalence was estimated for 12-yr-olds using previous New Caledonian data [2] and for 6- and 9-yr-olds, from a French study which was conducted under the same conditions [3]. The desired sample size was 300 for each 6- and 9-yr-old groups and 400 for 12-yr-olds. The sample size was adjusted to take into account a cluster effect (1+ (m-1)*ICC) with m = 24 being the mean number of children per classroom and an Intra-Class Coefficient (ICC) of 0.05 estimated from data of a previous survey [3] and a participation rate >75%. Finally, the number of children to be selected was set at a minimum of 800 at age 6 and 9 years and 1100 at age 12 years.

### Sampling method

Children were randomly selected using a computerised cluster sampling method (cluster  =  school) with a probability proportional to size [4], [5]. The pool of NC schools (217 public and 107 private) was stratified according to the area (South/North/Island) and the type of school (public/private). Seventy-six schools were selected. All the children aged 6 and 9 years in 2011 (40 public schools and 16 private; 14 in the North region, 32 in South and 10 in Islands) and 12 years in 2012 (10 public schools and 10 private; 6 in the North region, 9 in South and 5 in Islands), were included in the study. The total sample size was 3138 children (911 6-yr-olds, 923 9-yr-olds and 1304 12-yr-olds).

### Ethical concerns

In the absence of a single New Caledonian ethical committee, ethical approvals were obtained from the New Caledonian educational institutions (Evangelical Alliance for School Education (ASEE), Protestant federation for education (FELP), Diocesan Directorate for Catholic Education (DDEC), Directorate for education in South (DES), Directorate for youth, education, training and integration (DEFIJ), Directorate for education in Islands (DEPIL), New Caledonian directorate for education (DENC), New Caledonian Vice Rectory) and from the New Caledonian health authorities (Directorate for community actions and health in Islands (DACAS), Directorate for health and social affairs and of social problems in northern province (DASSPS-Nord), Directorate for sanitary and social action of southern province (DPASS-Sud), Sanitary and Social Agency of New Caledonia (ASSNC), Directorate of health and social affairs of New Caledonia (DASSNC). Schools were approached through local educational authorities. Explanatory letters and consent forms were sent to parents a few days prior to the dental examinations and those children whose parents returned written consent were examined.

### Study variables and health indicators

Pertinent indicators were identified through a literature review and after interviews with professional experts in epidemiology and paediatric dentistry. The children's dental status was evaluated during the clinical examination.

Dental caries was diagnosed at the threshold level (D3) for deciduous and permanent teeth, and at the enamel threshold level (D1) for the first permanent molars [6]. For caries experience, five indices were recorded: (1) numbers of decayed deciduous and permanent teeth (d3t, D3T), (2) numbers of decayed or filled deciduous teeth (d3ft), (3) numbers of decayed, missing or filled permanent teeth (D3MFT), (4) numbers of decayed first permanent molars (D1M), (5) numbers of decayed, missing or filled first permanent molars (D1MFM). Additionally, the significant caries index, which is the mean number of decayed, missing or filled permanent teeth for the third of the subjects having the highest D3MFT values, was calculated [7]. Radiographs were not used. The Gingival Index of Löe & Silness [8] was used to express the gingival status, and the scores were dichotomised: without gingivitis (score 0 for all sextants) or with gingivitis (score >0 for at least one sextant). Chewing efficiency was evaluated by recording the number of functional dental units (PFU). It has been demonstrated that the number of PFU measured clinically is a pertinent and discriminant criterion that independently influences nutritional status in adults [9]–[11]. The relationship between PFU and orofacial dysfunctions in children is being investigating: firsts results indicate that is it a good indicator of masticatory performance in children too [12]. The presence of at least one dental sealant on a permanent molar and at least one discoloured permanent tooth or non-carious dental structural defect were also recorded.

Socio-demographic variables (gender, date of birth, region of residence, public or private school) were recorded from the school register. Socioeconomic variables (type of health insurance and father's or mother's employment status) were recorded only for 6- and 9-yr-olds by school managers. Additionally, children responded to questions relating to their lifestyle (frequency of tooth brushing, daily consumption of sweet drinks), dental attendance (had never visited a dentist or had already visited a dentist) and dental fear (VAS scale from 0, no fear, to 10, severe fear) [13]. The children' self-perceptions of oral health (has no oral health problem or has oral health problems) were recorded for 9- and 12-yr-olds and the self-perception of chewing difficulties (can bite or chew every food item or type of food, difficult to bite or chew, or some types of food impossible to bite or chew) and smoking habits (had never smoked, had already smoked) were recorded for 12-yr-olds only. Finally, at age 12 yr, children were asked to which main cultural or ethnic group (Kanak/Polynesian/Caledonian/European, Asian/Other) they felt they belonged. A detailed list of the initials and acronyms is given Table 1.

**Table 1:** Description of the abbreviations and acronyms.

dt	Number of deciduous teeth with at least one carious lesion
DT	Number of permanent teeth with at least one carious lesion
dmft	Number of decayed, missing and filled deciduous teeth
DMFT	Number of decayed, missing and filled permanent teeth
DM	Number of first permanent molars with at least one carious lesion
DMFM	Number of decayed, missing and filled first permanent molars
SiC Index	Mean DMFT of the one third of the study group with the highest caries score for 12 years old children [7].
D_3_	Dentine threshold for carious lesions according to the Ekstrand classification [6]
D_1_	Enamel threshold for carious lesions according to the Ekstrand classification [6]
PFU	Posterior Functional Unit: pair of antagonist post-canine teeth having at least one occlusal contact
DF-VAS	Dental fear-Visual analogic scale
SES	Socio-Economic Status
CI	Confidence Interval
AMG	Aide Médicale Gratuite (Free Medical Assistance)
OHP	Oral Health Promotion
WHO	World Health Organization

### Investigators and Calibration

Seven general dental practitioners performed the children's examinations. They were recruited through an email announcement delivered by the local dentists' registration authority. Examiners were assigned to one or several schools, depending on feasibility criteria that were mostly depending on geographical locations. Children from the same school or the same grade were not systematically examined by the same investigator. Prior to the start of the study, all the investigators underwent a two-day training course consisting of projection of pictures illustrating clinical situations and explaining the data collection process. This training method has been described in a previous study [14]. The investigators were subjected to a test-retest procedure within a one-month period in order to check their reliability for the main study variables (D_3_T, d_3_t). Re-examining ten children for each examiner allowed intra-examiner reliability to be assessed. Inter-examiner reproducibility was evaluated using photographs illustrating various clinical situations for the study variables. Intra- and inter-examiner agreements were evaluated using Cohen's Kappa and varied from 0.46 to 0.92 and from 0.69 to 1, depending on the clinical cases.

### Data collection

In the 6-yr-old and 9-yr-old groups, children answered questions during a personal interview with an investigator. For the 12-yr-old children, the questionnaire was self-administrated. Children of all groups were examined while seated facing the examiner either in the school dispensary or in a classroom. For the oral examination, investigators used disposable gloves and mouth mirrors and a portable light. Probing was not permitted but debris could be removed with cotton rolls, if necessary. To count functional units (PFU), examiners used blue articulating paper of 200 µm thickness (Bausch) [15]. The children were asked to bite on the paper and the number of posterior mandibular teeth that had at least one coloured mark was counted.

### Data analysis

Data entry was duplicated and errors were corrected on Microsoft ACCESS software before analysis. The statistical analyses were performed using the Statistical Package for the Social Sciences (SPSS, Inc. Chicago, IL, USA, version 16.0) and Stata software (version 13, Stat/SE, StataCorp, College Station, US). To describe the dental status of Caledonian children (national epidemiological survey), all statistical analyses were conducted separately for each age group as participants were stratified by age. Results were expressed with 95% confidence intervals (CI). According to literature [16]–[19], mixed models were used, including the school and examiner parameters as random effects [20]. Indeed, due to the correlation of observations within a school, the usual tests were invalid and inter-cluster variances were inflated. Moreover, the number of examiners was high and despite the calibration process, an examiner effect was expected. The examiner effect was integrated throughout the analysis because it differed from the school effect and was significant in the South Region. Carious status was explained using explanatory variables following two steps, firstly with a bivariate analysis and then with a multiple random-effects regression. Factors significantly associated with d3t and D3T in bivariate analyses were entered in the regression models if p<0.1. Non-significant parameters considered as being clinically relevant were maintained in the analyses (sex, region, type of school, frequency of tooth brushing, consumption of sweet drinks and dental sealants) taking into account variability between and within cluster and examiner [21]. The mean numbers of carious lesions on deciduous teeth (d3t) or permanent teeth (D3T) were not normally distributed (even with a normality transformation), thus a log transformation was used. Chi-squared tests were used to study the interrelationships between oral health variables. The interactions between factors were tested and the results were reported using regression coefficients, 95% confidence intervals (CI) and P-values (P). The level of significance (type I error α) was set at 0.05.

## Results

### Sample description

Of the children initially selected, 87% participated in the study; 2734 children were thus examined and interviewed (744 6-yr-olds, 789 9-yr-olds and 1201 12-yr-olds). The variables age, sex, area and type of school had similar distributions in the sample and in the study population. Among the participants, 52% were boys, >70% of the children attended public schools, 10% lived in the Islands area, 20% in the northern and 70% in the southern region. Among the 12 yr-old group, 45% of children saw themselves as Caledonian, 27% Kanak, 18% Polynesian, 9% European or Asian or other communities and 3% did not want to answer the question (Table 2).

**Table 2:** Subjective oral health, social and behavioural characteristics of the studied population.

	Age 6 yr		Age 9 yr		Age 12 yr	
	*N*	%	*N*	%	*N*	%
**Gender**	*744*		*789*		*1201*	
Male		52.1		51.8		51.8
Female		47.9		48.2		48.2
**Type of school**	*744*		*789*		*1201*	
Public		69.9		76.2		74.8
Private		30.1		23.8		25.2
**Region of Residence**	*744*		*789*		*1201*	
Islands		10.3		10.1		9.2
North		18.7		21.2		20.7
South		71.0		68.7		70.1
**Cultural or Ethnic group**	NI		NI		*1161*	
Kanak						27.3
Polynesian						18.2
Caledonian						45
European, Asian and others						9.5
**Mother's employment status**	*509*		*505*		NI	
Unemployed		29.5		42.6		
blue collar, tradesman		32.0		20.6		
Management, executive		38.5		36.8		
**Father's employment status**	*479*		*485*		NI	
Unemployed		10.2		9.9		
blue collar, tradesman		59.7		49.1		
Management, executive		30.1		41.0		
**Type of health insurance**	*508*		*508*		NI	
Basic insurance only		22.8		20.1		
State aid supplemental (AMG)		26.8		33.1		
Private supplemental		50.4		46.8		
**Usual drinks when thirsty**	*741*		*789*		*1199*	
Water		69.6		64.9		64.6
Sweet drinks		30.4		35.1		35.4
**Smoking habits**	NI		NI		*1201*	
Never smoked						88.3
Has smoked						11.7
**Frequency of tooth brushing**	*739*		*789*		*1196*	
<1 weekly		25.6		37.5		20.2
≥1 weekly		8.1		6.7		24.5
≥1 daily		66.3		55.8		55.3
**Dental attendance**	*730*		*785*		*1189*	
Had never visited a dentist		49.7		22.0		16.9
Had visited a dentist		50.3		78.0		83.1
**Dental fear (VAS scale from 0 (no fear) to 10 (severe fear))**	*739*		*780*		*1201*	
DF-VAS 0–3		77.7		61.1		64.6
DF-VAS 4–6		11		23.2		19.2
DF-VAS 7–10		11.3		15.7		16.2
**Self-perception of oral health**	NI		*687*		*1092*	
No oral health problem				41.2		50.9
Oral health problems				58.8		49.1
**Chewing difficulties**	NI		*NI*		*1011*	
Food impossible to chew						16.7
Food difficult to chew						14.6
No difficulties						68.6

N: Numbers; %: Proportion; NI: not investigated.

### Socioeconomic status

Data about socioeconomic status was missing in more than 30% of children. Unemployment was approximately 10% for fathers, and varied from 29% to 43% for mothers, depending on age group. More than 20% of the 6-yr-old and 9-yr-old children were covered by the basic compulsory health insurance alone; 27% of the 6-yr-olds and 33% of the 9-yr-olds benefited from a state aid supplemental insurance dedicated to economically disadvantaged families (Table 2).

### Lifestyle and experience of dental care

Negative dental behaviours were noticed for some children; more than 30% of the children consumed sweet drinks regularly, almost 12% of the 12-yr-olds had already smoked and more than one third of the children did not brush their teeth daily (Table 2).

In the study sample, half of the 6-yr-old children had already attended a dentist and this proportion rose to 83% for 12-yr-olds. Dental fear varied slightly by age; older children tended to have higher scores and more than 15% of the 9- to 12-yr-olds had high scores (DF-VAS >6). Almost 60% of the children at age nine yr and 50% of the children at age 12 yr considered that they had oral health problems and more than 30% of the 12-yr-olds reported having chewing difficulty (Table 2).

### Dental status

Data on dental status are gathered in Tables 3 and 4. More than 50% of the children had poor oral hygiene indicated by gingivitis and 12-yr-olds were more affected by gingivitis than younger children. At age six yr, 58% had at least one untreated carious lesion (d_3_t or D_3_T >0) and the mean d_3_t was 2.58 (±3.40) teeth. The prevalence of untreated carious lesions in the mixed dentition was high at age nine yr (60%) with a mean D_3_T of 0.53 (±1.04) teeth. At age 12 yr, 47% of the children had at least one untreated carious deciduous or permanent tooth and the mean D_3_T was 1.49 (±2.39) teeth and one third of the most affected children had 5.37 (±2.80) teeth with at least one carious lesion. The number of permanent first molars affected by initial or dentinal carious lesions increased with age, from D_1_M of 0.24(±0.64) teeth among 6-yr olds to D_1_M of 0.84 (±1.11) teeth in 12-yr-olds.

**Table 3:** Prevalence of oral diseases.

	Age 6 yr		Age 9 yr		Age 12 yr	
Oral status	*N*	% [95%CI]	*N*	% [95%CI]	*N*	% [95%CI]
**Caries**	*744*		*789*		*1201*	
No carious lesion (d_3_t or D_3_T = 0)		42.3 [38.8;46.0]		40.1[36.6;43.6]		53.1[50.3;56.0]
At least one carious lesion (d_3_t or D_3_T>0)		57.7 [54.0;61.2]		59.9[56.4;63.4]		46.9[44.0;49.7]
**Gingival status**	*731*		*787*		*1201*	
No gingivitis		46.9 [43.3;50.6]		40.4[37.0;43.9]		37.8[35.1;40.6]
Gingivitis on one or more sextants		53.1 [49.4;56.7]		59.6[56.1;63.0]		62.2[59.4;64.9]
**Number of posterior Functional units**	NI		*754*		*1201*	
<4 PFU				15.1[12.6;17.9]		2.7[1.9;3.8]
4 to 6 PFU				43.1[39.5;46.7]		17.0[14.9;19.2]
>6 PFU				41.8[38.2;45.4]		80.3[77.9;82.5]
**Dental structure of permanent teeth**	*740*		*787*		*1198*	
No structural defect		85.6[82.8;88.0]		74.6[71.4;77.6]		80.0[77.6;82.2]
At least one structural defect		14.4[12.0;17.2]		25.4[22.4;28.6]		20.0[17.8;22.4]

N: Number; %: Proportion; NI: not investigated; CI: confidence interval.

**Table 4:** Dental status and caries experience (Mean ± standard deviation).

	Age 6 yr	Age 9 yr	Age 12 yr
	*N = 744*	*N = 789*	*N = 1201*
Number of Permanent teeth	4.61±3.51	15.87±4.19	25.74±3.13
Number of Deciduous teeth	16.87±2.82	7.35±4.26	0.68±1.77
D_3_MFT	0.09±0.44	0.76±1.29	2.09±2.82
D_3_T	0.07±0.36	0.53±1.04	1.49±2.39
d_3_ft	3.01±3.56	2.26±2.17	0.95±1.16
d_3_t	2.58±3.4	1.72±1.99	0.73±1
SiC Index	NA	NA	5.37±2.80
D_1_MFM	0.33±0.82	1.70±1.53	1.92±1.59
D_1_M	0.24±0.64	1.06±1.24	0.84±1.11
Number of sealed molars	0.11±0.53	0.39±0.95	0.32±0.95

N: Number; NA: not applicable.

The carious components (d_3_t, D_3_T and D1M) were predominant within the global components of caries experience (d_3_ft, D_3_MFT and D1MFM), indicating that dentinal carious lesions remained mostly untreated. The mean number of sealed permanent molars was also very low (fewer than one) in all age groups.

Fifteen per cent of the 9-yr-olds had fewer than four functional units but this proportion was lower at age 12 yr (2.7%). The proportion of children with enamel or dentinal defects on permanent teeth was high ( >14%), particularly in 9-yr-old children (25%), but the type of defect was not specified.

### Inter-relationships between oral health problems

Infectious oral health problems were inter-related in all age groups, with a higher number of carious lesions in children with gingivitis (Chi-squared test, P<0.0001).

### Risk factors related to caries experience

Caries experience varied substantially depending on socioeconomic status, environment and culture. Six yr-old children with the state aid dental insurance and children at age six and nine yr whose mothers were jobless had more untreated carious teeth compared with other children. On the other hand, children in public schools had less untreated dental caries than children attending private schools in the 6-yr-old group. In the 12-yr-old group, the mean number of untreated carious teeth was higher among Kanak, Polynesian and Caledonian children when compared with European and Asian children. It also appeared that 6-yr-old children living in the Islands had more untreated dental caries (Table 5).

**Table 5:** Bivariate analysis: relationship between caries experience (d_3_t, D_3_T) and environmental or behavioural variables.

	Age 6 yr			Age 9 yr			Age 12 yr		
	N	d3t+	p*	N	D3T	*p*	N	D3T	*p*
**Gender**	724		*Ns*	789		*ns*	1201		*ns*
Male		2.6 (3.5)			0.51 (1)			1.38 (2.1)	
Female		2.55(3.3)			0.55 (1.1)			1.6 (2.6)	
**Type of school**	743		*<0.05*	789		*ns*	1201		*ns*
Public		2.41 (3.3)			0.54 (1)			1.4 (2.3)	
Private		3.02 (3.5)			0.5 (1)			1.73 (2.5)	
**Region**	743		*<0.05*	789		*ns*	1201		*ns*
Islands		4.06 (3.3)			0.57 (1)			1.46 (1.8)	
North		2.39 (3)^a^			0.35 (0.7)			1.82 (2.6)	
South		2.43 (3.5)^a^			0.58 (1.1)			1.39 (2.4)	
**Cultural or Ethnic group**	NI			NI			1161		*<0.01*
Kanak								1.58 (2.4)	
Caledonian								1.64 (2.4)	
Polynesian								1.56 (2.7)	
European, Asian and others								0.45 (1.3)^a^ ^, b, c^	
**Mother's employment status**	509		*<0.05*	505		*<0.05*	NI		
Unemployed		3.44 (3.9)			0.69 (1.1)				
Blue collar, tradesman		2.67 (3.3)			0.36 (0.8)^a^				
Management, executive		1.9 (3)^a^			0.44 (0.9)^a^				
**Father's employment status**	479		*ns*	485		*<0.01*	NI		
Unemployed		3.26 (4.4)			0.44 (0.9)				
Blue collar, tradesman		2.74 (3.3)			0.66 (1.1)				
Management, executive		1.96 (3.2)			0.41 (1)^b^				
**Type of health insurance**	508		*<0.001*	508		*ns*	NI		
State aid supplemental		3.5 (3.5)			0.49 (1)				
Basic insurance only		3.26 (3.8)			0.63 (1.2)				
Private supplemental		1.8 (2.9)^a^ ^, b^			0.42 (0.8)^b^				
**Usual drinks when thirsty**	740		*<0.05*	789		*ns*	1199		*<0.05*
Sweet drinks		2.96 (3.5)			0.56 (1.1)			1.74 (2.45)	
Water		2.42 (3.3)			0.5 (1)			1.35 (2.3)	
**Smoking habits**	NI			NI			1139		*<0.01*
Has smoked								2.16 (2.8)	
Never smoked								1.39 (2.2)	
**Frequency of tooth brushing**	729		*ns*	789		*<0.01*	1173		*<0.001*
<1/day		3.01 (3.4)			0.73 (1.2)			1.93 (2.5)	
≥1/day		2.35 (3.4)			0.37 (0.8)			1.11 (2.1)	
**Dental attendance**	730		*ns*	785		*ns*	1189		*Ns*
Had never visited a dentist		2.6 (3.4)			0.5 (1)			1.34 (2.1)	
Had already visited a dentist		2.6 (3.4)			0.53 (1.1)			1.51 (2.4)	
**Dental sealants on permanent molars**	593		*ns*	789		*<0.001*	1201		*<0.001*
Absence of dental sealants		2.68 (3.4)			0.58 (1.1)			1.64 (2.5)	
At least one sealant		2.45 (2.5)			0.32 (0.7)			0.57 (1.3)	
**Dental Fear-Visual Analogue Scale**	738		*ns*	780		*ns*	1201		*<0.01*
DF-VAS <3		2.5 (3.4)			0.52 (1)			1.39 (2.3)	
DF-VAS 3-6		2.86 (3.8)			0.49 (1)			1.42 (2.39)	
DF-VAS>6		2.95 (3)			0.62 (1.1)			1.92 (2.5)^a^ ^, b^	
**Self-perception of oral health**	NI			687		*<0.05*	1092		*<0.001*
No problem					0.35 (0.8)			1.04 (1.8)	
Oral health problems					0.66 (1.2)			1.94 (2.8)	
**Chewing difficulties**	NI			NI			1011		*<0.05*
Food impossible to chew								1.81 (2.4)	
Food difficult to chew								1.57 (2.2)	
No difficulties								1.36 (2.3)^a^	
**Number of posterior functional units (PFU)**	NI			754		*<0.001*	1201		*Ns*
PFU<4					0.94 (1.2)			1.78 (3.8)	
4≤PFU<6					0.63 (1.1)^a^			1.32 (2.1)	
PFU≥6					0.33 (0.8)^a^ ^, b^			1.51 (2.4)	

N: Number; NI: not investigated; ns: not significant

+ Mean (Standard deviation), * p value

a p<0.05 vs 1^st^ modality, ^b^ p<0.05 vs 2^nd^ modality and ^c^ p<0.05 vs 3^rd^ modality

Caries experience was significantly related to the children's lifestyle and their access to preventive dental care. Indeed, six and 12-yr-old children who used sweet drinks daily and 12-yr-olds who had already smoked had higher caries scores than others. A higher number of decayed teeth was also observed for children who reported they did not brush their teeth daily at ages nine yr and 12 yr. In all groups, dental status did not differ, whether or not a child had already been to the dentist, but 9- and 12-yr-old children who had benefited from preventive dental care (with dental sealants) had less caries experience than others (Table 5).

Children's dental status was associated with dental anxiety, perceptions about their own oral health and children's oral functioning. At age 12 yr, children with lower levels of dental fear (DF-VAS<3) had less caries experience than children with higher DF-VAS levels (DF-VAS >6). Moreover, nine and 12-yr-old children who reported having no oral health problems and 12-yr-olds who reported they had no difficulties for chewing had better dental status than others. Nine yr-old children with fewer untreated carious lesions had significantly more functional units (PFU) than other children (Table 5).

### Multidimensional analysis

All the variables, which were significantly related to caries experience in the bivariate analyses, were included in the model. The variables gender, region, type of school were included in the model even if no significant relationship with dental status was observed in the bivariate analysis. Table 6 gives the regression coefficients only for the explanatory variables that were significant for at least one age group.

**Table 6:** Regression coefficients derived from regression: Caries experience (d_3_t, D_3_T) as dependent variable and environmental or behavioural risk factors as independent variables.

	Age 6 yr *(N = 744)*			Age 9 yr *(N = 789)*			Age 12 yr *(N = 1201)*		
	d_3_t			D_3_T			D_3_T		
	RC	95% CI	*p*	RC	95% CI	*p*	RC	95% CI	*P*
**Type of health insurance**							NI		
State aid									
Basic	0.59	−0.43 1.62	*ns*						
Private	−1.02	−1.99–0.05	*<0.05*						
**Frequency of tooth brushing**									
≥1/day vs <1/day				−0.34	−0.56 −0.13	*<0.01*	−0.35	−0.64 −0.05	*<0.05*
**Usual drinks when thirsty**									
Sugared drinks vs water	0.76	0.02 1.52	<0.05						
**Cultural or Ethnic group**	NI			NI					
Kanak									
European, Asian and others							−0.79	−1.35 −0.24	*<0.01*
Caledonian							−0.02	−0.57 0.16	*ns*
Polynesian							−0.23	−0.68 0.22	*ns*
**Dental sealants on permanent molars**									
At least one sealant vs Absence							−1.10	−1.50 −0.70	*<0.001*
**Self-perception of Oral Health**	NI								
Oral health problems vs No problem							0.68	0.42 0.95	*0.001*
**Number of posterior functional units (PFU)**	NI								
PFU<4									
4≤PFU<6				−0.30	−0.62 0.02	*ns*			
PFU≥6				−0.51	−0.84 − 0.18	*<0.01*			
**Chewing difficulties**	NI								
Food impossible to chew									
Food difficult to chew							−0.43	−0.91 0.05	ns
No difficulties							−0.42	−0.80 −0.35	*<0.05*

Other non-significant variables included in the models were not presented here. These were: for the 6 yrs group: sex, region, type of school, mother employment status; for the 9 yrs group: sex, region, type of school, mother and father employment status; for the 12 yrs group: sex, region, type of school, dental fear, smoking status.

N: Number, CI: confidence interval, NI: not investigated, ns: not significant

The multidimensional random effects model showed a significant influence of the type of health insurance, cultural background, oral hygiene, consumption of sugared drinks and dental preventive care on dental caries experience (Table 6). In six yr-olds, the type of health insurance and the consumption of sweet drinks significantly influenced the number of carious lesions. In 9 and 12-yr-olds, frequency of tooth brushing and presence of dental sealant also showed a significant association with the number of carious lesions. When compared with the Kanak 12-yr-olds, Europeans and Asians had fewer carious lesions (Table 6).

On the other hand, significant associations were found between lower numbers of carious lesions, a high number of functional units and no perception of oral health problems. Nine yr-old children with more than 6 PFUs were significantly less affected by dental caries than children with fewer than 4 PFUs and 12-yr-olds who reported that they had no oral problems had fewer carious lesions. Moreover, perception of chewing difficulties by 12 years old children was related to the number of carious teeth (Table 6).

## Discussion

This survey aimed to evaluate the dental status of children at age six, nine and twelve years old in New Caledonia. The participation rate was high; 2734 children were examined. In the study sample, more than half of the children were affected by gingivitis and the prevalence of untreated dental caries was almost 60% among 6- and 9-yr-olds and about 50% among 12-yr-olds. Caries experience is unevenly distributed in the population, with one third of 12-yr-olds having more than five teeth with at least one carious lesion. Dental caries experience was particularly high in socially deprived children, those who had an unfavourable lifestyle or who had not benefited from preventive dental care. The presence of a high number of untreated carious lesions had a negative impact on the children's perceptions of their oral health and the number of functional dental units

The dental status of children has largely improved since the last epidemiological survey was conducted in New Caledonia in collaboration with the WHO in 1996 [2]. The mean D_3_MFT at age 12 yr has decreased from 4.09 teeth in 1996 to 2.09 teeth in 2012. This decline has been observed all around the world in developed countries over recent decades. The evolution is mainly attributed to changes in living conditions, lifestyles and improved self-care practices including the effective use of fluorides [1], [22], [23]. The disease has not been totally eradicated, but is controlled to a certain degree by retarding the initiation of carious lesions to a later age, or totally preventing the condition [1].

According to the "2003 World Oral Health Report", the current dental status of 12-yr-olds in New Caledonia is similar to that observed in other developed countries such as the USA (D_3_MFT = 3.0) or Europe (D_3_MFT = 2.6) [23], [24]. For 9-yr-olds, the same observation can be made with similar caries experience levels as in the present study and in a French region in 2006 (respectively D_3_MFT = 0.76 and 0.85) [3]. Greater improvement could be achieved for the youngest New Caledonian children. The prevalence of dental caries in the mixed dentition remains high in the New Caledonian 6-yr-olds (58%) when compared with that observed in a group of 6-yr-old French children (33%) examined in 2009 [25]. Important disparities were found in the present study, with high caries burden in children with unfavourable health behaviours, deprived social background, low access to preventive dental care or from the Kanak culture. The association of oral health with social or cultural determinants is well known, in France and in other countries [3], [26]–[28]. In New Caledonia, such inequalities are particularly noticeable.

The present study has also shown that the weight of the D component of the DMFT and DMFM was very high in all age groups, while the relative weight of the F and M components were weak. Moreover, the number of untreated carious lesions did not decrease for children who had already visited a dentist. It is thus suggested that the dental care on offer is not in accordance with the population's needs. Three actors are involved in the care delivering: the children, the parents and the practitioners. As shown in a previous French study, children with anxiety about dental care were more affected by dental caries [29], however this study did not explore the relationship between dental anxiety and dental care distribution in NC. Parental social deprivation and low education are often involved as barriers for children's dental treatment. In New Caledonia, per capita income is high and the whole population is covered by basic public medical and dental insurance with a supplemental state subsidised insurance for low income families. Nevertheless, there is no comprehensive, free of charge public dental service for children as health services in schools. Moreover, as in many other countries, dentistry in New Caledonia uses a patient-centred curative approach. Even when dental services are available at no charge, other barriers to care may exist [27]. Socially deprived populations and ethnic minorities are sometimes reluctant to consult dentists because of previous negative experience, communication barriers and ignorance of their rights. They also perceive oral health in a way that strongly influences their treatment preferences and explains low and selective use of dental services [30]–[33]. This study did not explore the practitioner-related barriers for NC children's dental care. Further approaches are required to solve the problem of dental care access for children with high dental needs and deprived social backgrounds [34], [35].

The study has some limitations. The sample size and the population's geographical spread made the enrolment of seven examiners necessary. To minimize the limitations of this methodological concern, the inter- and intra-examiner variability was limited by thoroughly calibrating the examiners. A moderate to very good level of agreement was obtained, depending on the examiner pair. Furthermore, a mixed model was used for all the statistical comparisons, which treated the cluster and examiner parameters as random effects. These methodological and statistical precautions prevented our results from being highly biased by this parameter.

The second limitation of our study is that some variables were recorded using a questionnaire completed by the school managers. Several reminders were sent to the non-responding schools but only 80% of the questionnaires were returned. Despite this non-response rate (some families may not have wanted to give personal information about their socioeconomic status), the final sample remained representative of the study population in terms of gender, region and type of school attended. Thus, we could reasonably assume that the non-responses were not linked to specific variables and had not reduced the quality of our results.

The third limitation is the non-inclusion of children with disabilities in the study sample due to the lack of demographic information on children with disabilities living at home, attending ordinary schools or living in educational institutions. However, data on oral health condition of this population are necessary to elaborate a fair comprehensive oral health programme, aiming to reduce inequalities in oral health. Consequently a further survey in special schools is needed, as has been undertaken in France [36].

In conclusion, this study highlights the need for new strategies aimed at improving oral health and at reducing inequalities. An alternative approach is needed, based on the principles of oral health promotion (OHP) such as those developed in the WHO's Ottawa Charter [37]. The development of healthy environments and the adoption of health promoting behaviours would be particularly suited for schools and the collaboration with local populations would probably facilitate improvements in children's dental health, as in the health promoting schools programs [38]. OHP programs, with culturally relevant interventions for the populations most concerned with oral diseases, and multidisciplinary collaborative networks also need to be developed. A reorientation of dental services towards preventive interventions and fewer operative treatments would be desirable. An OHP program would need to be developed in connection with other health programs in New Caledonia, namely programs related to the prevention of other chronic diseases as rheumatic heart disease or obesity. The common risk factor approach would need to be used in order to address risk factors common to those chronic conditions within the context of the local environment.
